# Detection of persistent SARS-CoV-2 IgG antibodies in oral mucosal fluid and upper respiratory tract specimens following COVID-19 mRNA vaccination

**DOI:** 10.1038/s41598-021-03931-3

**Published:** 2021-12-27

**Authors:** Aubree Mades, Prithivi Chellamathu, Noah Kojima, Lauren Lopez, Melanie A. MacMullan, Nicholas Denny, Aaron N. Angel, Marilisa Santacruz, Joseph G. Casian, Matthew Brobeck, Nina Nirema, Jeffrey D. Klausner, Frederick Turner, Vladimir I. Slepnev, Albina Ibrayeva

**Affiliations:** 1 Curative Inc., San Dimas, CA 91773 USA; 2 Department of Medicine, University of California Los Angeles, Los Angeles, CA 90095 USA; 3 Mork Family Department of Chemical Engineering and Materials Science, Viterbi School of Engineering, University of Southern California, Los Angeles, CA 90089 USA; 4 Department of Preventive Medicine, University of Southern California Keck School of Medicine, Los Angeles, 90089 USA; 5 Eli and Edythe Broad Center for Regenerative Medicine and Stem Cell Research at USC, Department of Stem Cell Biology and Regenerative Medicine, W.M. Keck School of Medicine, Los Angeles, USA; 6 Davis School of Gerontology, University of Southern California, Los Angeles, CA USA; 7 Curative, Inc., 605 East Huntington Drive, Monrovia, CA 91016 USA

**Keywords:** Infectious diseases, Viral infection, Epidemiology

## Abstract

COVID-19 mRNA vaccines are highly effective at preventing COVID-19. Prior studies have found detectable SARS-CoV-2 IgG antibodies in oral mucosal specimens of participants with history of COVID-19. To assess the development of oral SARS-CoV-2 IgG antibodies among people who received either the Moderna or Pfizer/BioNTech COVID-19 vaccination series, we developed a novel SARS-CoV-2 IgG enzyme-linked immunosorbent assay (ELISA) to quantify the concentrations of oral and nasal mucosal SARS-CoV-2 IgG levels. We enrolled 52 participants who received the Moderna vaccine and 80 participants who received the Pfizer/BioNTech vaccine. Oral mucosal specimens were self-collected by participants prior to or on the day of vaccination, and on days 5, 10, 15, and 20 following each vaccination dose and 30, 60, and 90 days following the second vaccination dose. A subset of the cohort provided additional nasal mucosal specimens at every time point. All participants developed detectable oral mucosal SARS-CoV-2 IgG antibodies by 15 days after the first vaccination dose. There were no significant differences in oral mucosal antibody concentrations once participants were fully vaccinated in the Moderna and Pfizer/BioNTech vaccines. Oral or nasal mucosal antibody testing could be an inexpensive and less invasive alternative to serum antibody testing. Further research is needed to understand the duration of detectable oral or nasal mucosal antibodies and how antibody concentrations change with time.

## Introduction

The rapid development and administration of highly effective vaccines for COVID-19 is a major triumph^1^. Prior research has found that vaccines for COVID-19 not only drastically decrease risk of hospitalization and death, but also prevent risk of acquisition and transmission of COVID-19^2^. Mechanisms of protection against SARS-CoV-2 elicited from vaccines include humoral and cell-mediated immune responses^3^.

Vaccination against COVID-19 leads to the production of serum neutralizing antibodies^3^. Prior studies have found that serum antibodies by mRNA-1273 persisted for at least 6 months following the recommended second vaccination dose^4^. Earlier work from our group demonstrated that an ELISA-based qualitative assay can reliably detect the presence of SARS-CoV-2 IgG antibodies targeting spike proteins S1 and S2 from self-collected oral mucosal specimens among participants previously infected with SARS-CoV-2^5^. In the present study, we aimed to assess whether (1) SARS-CoV-2 IgG antibodies targeting the spike protein are detectable in self-collected oral and nasal mucosal specimens following COVID-19 mRNA vaccination; and (2) a quantitative assay could measure changes in SARS-CoV-2 IgG antibodies in oral and nasal mucosal fluid over time among vaccinated participants.

## Methods

Study enrollment was offered to healthcare workers at a COVID-19 testing site in San Dimas, California, USA, and interested healthy patients at vaccination sites in Riverside, California, USA and Round Rock, Texas, USA. Enrollment was offered to subjects 18 years of age and older. Initial recruitment took place among those who had received the first dose of the Moderna COVID-19 vaccine within the past 5 days. Later recruitment took place among those who had received the first dose of the Pfizer/BioNTech COVID-19 vaccine. Vulnerable subjects (pregnant persons, nursing home residents or other institutionalized persons, prisoners, and persons without decisional capacity) were not eligible for enrollment in this study. Verbal informed consent was obtained from each subject prior to enrollment in the study.

### Time points

Enrolled participants provided one self-collected oral fluid specimens for SARS-CoV-2 IgG antibody testing prior to or on the day of vaccination, as well as on days 5, 10, 15, and 20 following their first vaccination dose, and days 5, 10, 15, 20, 30, 60, and 90 following their second vaccination dose. Participants with a positive antibody test at the first time point or who missed more than one collection time point were excluded. A subset of the Pfizer/BioNTech cohort additionally provided a self-collected nasal swab fluid specimen for SARS-CoV-2 IgG antibody testing at every time point.

### Oral fluid specimen collection

At each time point, one sample was collected using the OraSure^®^ Technologies Oral Specimen Collection Device (OSCD; item number 3001-2870, OraSure^®^ Technologies, Bethlehem, PA) for SARS-CoV-2 IgG antibody detection), as previously described^2^. Participants were trained by study staff to self-collect specimens upon enrollment and observed during their first specimen collection. Self-collection involves scraping the pad of the OSCD for 5 s on all four quadrants of the inner gums and then placing the pad into the space between the cheek and one of the lower gums for 20 s to allow the pad to absorb fluid. Participants were asked not to eat, drink water, or brush their teeth for 30 min before performing the test. Participants were provided with additional test kits and asked to self-collect a specimen at each time point unobserved, with a 2-day grace period for sample collection and 2-day grace period for returning specimens to the study team. Specimens were left at room temperature for up to 5 days and then stored at − 80 °C until use.

### Anterior nares specimen collection

Anterior nares mucosal specimens were self-collected with a flocked nylon swab (CY-98000; Huachenyang Technology, China). The swab was rotated around each anterior nare for 15 s and then placed into the collected tube provided with the OSCD. Specimens were left at room temperature for up to 5 days and then stored at − 80 °C until use.

### Quantitative SARS-CoV-2 IgG ELISA assay

A SARS-CoV-2 IgG ELISA that provides semi-quantification was performed on self-collected oral and nasal mucosal specimens. To process the samples, 25 μL of sample diluent and 100 µL of either oral or nasal mucosa specimen were added to 96-well plates coated with both S1 and S2 subunits of the SARS-CoV-2 viral spike glycoprotein. Plates were incubated at ambient temperature for 1 h. Sample wells were then washed six times with wash buffer (20 × dilution with ddH2O, 350 µL per well) and conjugate solution was added (100 µL per well). Plates were incubated at ambient temperature for 1 h and sample wells were then washed an additional six times. Next, substrate solution was added (100 µL per well) and the plate was incubated at ambient temperature for 30 min. Finally, stop solution was added (100 µL per well). The absorbance of sample wells was measured immediately at 450 nm and 630 nm. Output reports generated the absorbance at 630 nm subtracted from the absorbance at 450 nm. All reagents and proteins were obtained from OraSure^®^ Technologies^2^.

To quantify SARS-CoV-2 IgG antibodies in oral and nasal mucosal samples, an S1-specific monoclonal IgG antibody with no known cross-reactivity to the S2 domain of the spike protein was used as a reference antibody. We confirmed lack of cross-reactivity from IgA and IgM with the absence of detectable IgA nor IgM signal on an assay. The standard curve was used to calculate the IgG antibody concentration in specimens from absorbance values at 450/630 nm from the ELISA assay. Specimens with antibody titer levels exceeding the range of the standard curve were diluted in a sample dilution buffer and re-ran. A standard curve was developed using a monoclonal IgG antibody targeting the S1 antigen of SARS-CoV-2 at concentrations of 0, 1.5, 3, 6, 12, and 20 ng/mL with a polynomial regression curve-fitting model. The absorbance signal from each sample is directly proportional to the IgG antibody concentration present in the oral fluid (Supp. Fig. 1). We determined the Limit of Detection (LOD) and Limit of Quantification (LOQ) per Clinical Laboratory Standards Institute guidelines^6^.

### Data analysis

All data analysis was performed using GraphPad Prism (GraphPad Prism Version 8.4.3) software. Paired, two-sided *t* tests were performed to compare differences in average antibody concentrations across conditions. All data can be made available upon reasonable request.

### Ethics

The study protocol was approved by the Advarra Institutional Review Board (IRB# Pro00048737). Because Curative is a private company that does not currently have an IRB, the Advarra IRB was selected to evaluate the protocol through recommendation by one of the authors of this manuscript. The Advarra IRB determined the protocol was minimal risk and verbal informed consent was sufficient for the research under 45 CFR 46.117(c). The study was performed in accordance with all policies, procedures, and regulations, as well as with all applicable federal, state and local laws regarding the protection of human subjects in research as stated in the approved IRB.

## Results

### Study cohort

In January 2021, 124 participants who had received the Moderna mRNA COVID-19 vaccine were enrolled. During the study period 68 participants were excluded from the study due to incomplete sample return and 5 were excluded due to a positive antibody test prior to vaccination. The resulting study cohort was comprised of 51 participants with an average age of 28.8 ± 8.2 years. Of the cohort, 32 (62.8%) participants identified as female.

In March 2021, 123 participants were enrolled the day they received the Pfizer/BioNTech mRNA COVID-19 vaccine. Oral fluid antibody specimens were collected from each participant directly after receiving vaccination. Of those participants, 36 were excluded from the study due to incomplete sample return and 14 due to a positive antibody test prior to vaccination, resulting in a 73 participant study cohort. The average participant age of this group was 49.8 ± 18.3 years. Of the cohort, 42 (57.5%) participants identified as female.

### Quantitative SARS-CoV-2 IgG antibody ELISA

The Limit of Detection (LOD) was 1 ng/mL and the Limit of Quantification (LOQ) was 1.5 ng/mL.

### Oral mucosal specimen antibody production following vaccination

**Figure 1:**
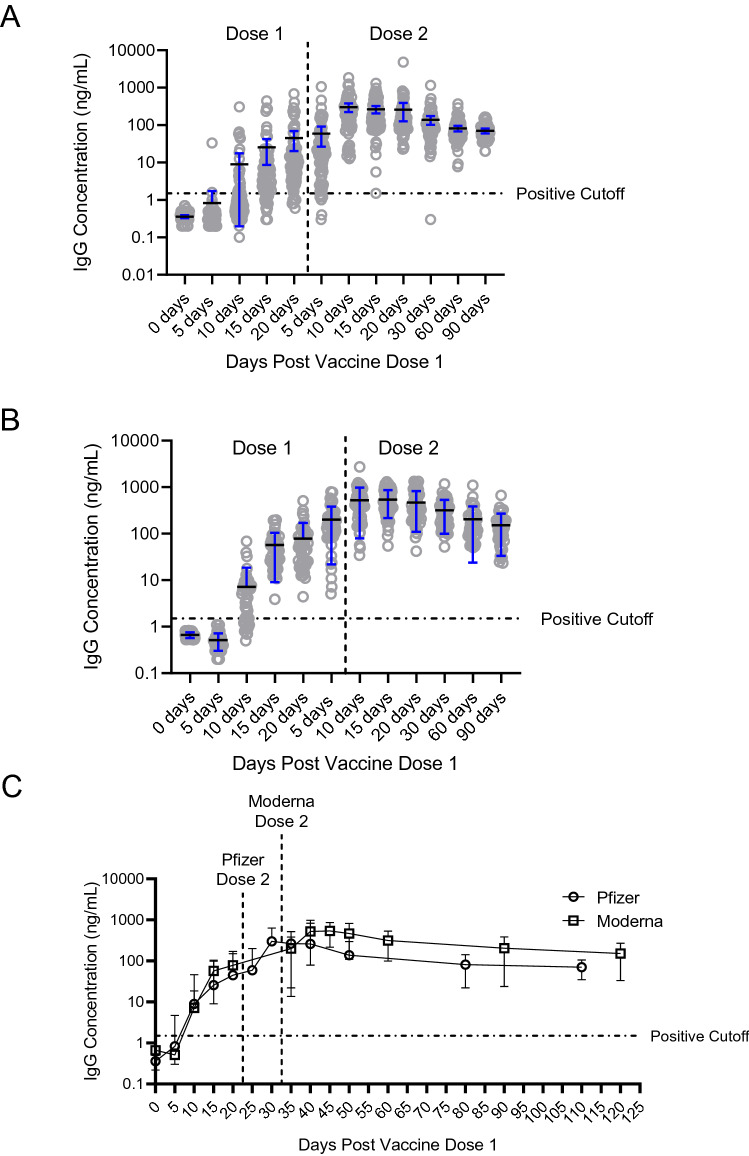
SARS-CoV-2 IgG antibody time course trajectories of participants receiving either Pfizer or Moderna vaccines were largely similar. (**A**) Relative quantification of SARS-CoV-2 IgG antibodies in participants who received the Moderna vaccine (N = 51) reveal increasing concentration in the days following the first and second dose. The average antibody concentration of participants prior to receiving vaccination was 0.66 ± 0.09 ng/mL while 90 days after vaccination this number stabilized at 151.7 ± 118.5 ng/mL. (**B**) Similarly, antibody concentration increased following vaccination by the Pfizer vaccine (N = 79). The average antibody concentration of participants prior to receiving vaccination was 0.35 ± 0.14 ng/mL while 90 days after vaccination this number stabilized at 70.32 ± 35.42 ng/mL. (**C**) Time courses of antibody development are well-aligned across the two vaccines.

**Figure 2:**
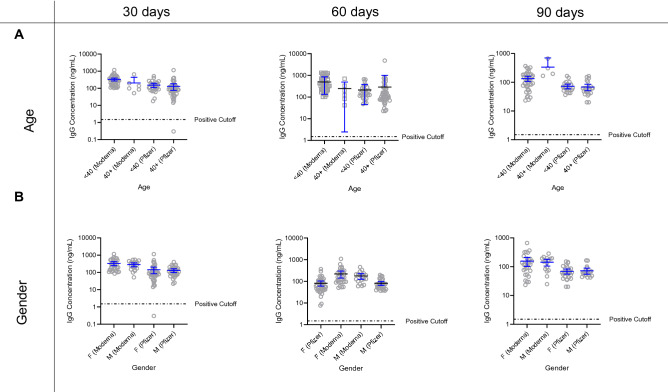
No significant correlation was found between demographics and relative antibody concentration in participants receiving either the Pfizer or Moderna vaccines 30, 60 or 90 days after receiving the second dose. (**A**) Age does not impact the concentration of antibodies conferred by vaccination. Age breakdowns of participants younger than age 40 and older than age 40 (inclusive) were used to determine that there is no statistical effect of age on antibody development post-vaccination. (**B**) Gender does not correlate to antibody concentration. In all participants vaccinated by either the Moderna or Pfizer vaccine, no statistical difference was observed based on gender.

Antibody concentrations in the two groups were not significantly different at 30, 60, or 90 days following the second vaccination dose (Fig. 1C). At 90 days following the second vaccination dose, the average IgG concentration in oral mucosa for Moderna participants (n = 44) was 151.7 ± 118.5 ng/mL (Fig. 1A), and for Pfizer/BioNTech participants (n = 47) was 70.32 ± 35.42 ng/mL (Fig. 1B). We did not find any statistically significant impact of either age or gender on antibody titers at 30, 60 and 90 days after receiving the second vaccine dose (Fig. 2).

### Paired oral and nasal mucosal specimen samples

Of participants that provided a paired oral and nasal mucosal specimen following their second vaccination dose (n = 28), 27/28 had detectable antibodies 15 days following their second dose, and 28/28 had detectable antibodies 30, 60 and 90 days following their second dose (Supp. Fig. 2).

## Discussion

We found that SARS-CoV-2 IgG antibodies can be detected and quantified in self-collected oral and nasal mucosal specimens following vaccination with a COVID-19 mRNA vaccine. All participants developed detectable SARS-CoV-2 IgG oral mucosal antibodies before receiving their second vaccine dose. The Moderna and Pfizer/BioNTech groups had similar antibody concentrations once participants were fully vaccinated.

Prior research conducted on the Moderna and Pfizer/BioNTech vaccines found that among adults who received a COVID-19 vaccination series, SARS-CoV-2 IgG antibodies were detected in serum samples by 15 days following the first vaccination dose^7–12^. However, to date there has been limited research on antibody detection in oral or nasal mucosa.

Our earlier work showed we can reliably detect SARS-CoV-2 IgG antibodies in oral mucosal specimens among participants previously infected with SARS-CoV-2.

To our knowledge, this is the first longitudinal study quantifying SARS-CoV-2 IgG antibodies in self-collected oral and nasal mucosal specimens following vaccination with a COVID-19 mRNA vaccine. IgG antibodies in oral and nasal mucosa may provide a possible biological explanation for the decreased rates of SARS-CoV-2 acquisition and severe COVID-19 disease among vaccinated individuals^13^. In ongoing studies, we aim to further understand how the antibodies developed in response to vaccination provide protection against SARS-CoV-2 variants of concern as well as the ancestral strain.

This study had limitations. Due to our enrollment of health care workers in the Moderna cohort, the average age of participants in this cohort was low, which limits generalizability. Participants were not monitored during specimen collection after the first time point, but the ability of participants to follow instructions for unobserved collection was validated internally prior to sample collection. We have not yet assessed non-mRNA-based COVID-19 vaccines. Additionally, the calculated values for oral fluid specimens presented in this paper represent the diluted specimen, with up to 800 µL of oral fluid diluted in 800 µL of preservative. The true oral fluid IgG antibody concentration may be up to several fold higher than the value reported, depending on the volume of fluid collected. Finally, it is not yet known how oral or nasal mucosal fluid antibody titers are directly comparable to serum titers for the protection of infection or disease.

## Conclusions

We found that SARS-CoV-2 IgG antibodies can be detected and quantified in self-collected oral and nasal mucosal specimens following vaccination with a COVID-19 mRNA vaccine. All vaccinated participants in this study developed detectable antibodies. There was no significant difference in antibody production between Moderna and Pfizer/BioNTech vaccine groups. This work provides a potential biological basis for protection and reduced transmission of SARS-CoV-2 among vaccinated people. Oral and nasal mucosal specimen testing may offer a less invasive method for antibody testing to blood collection. Further research is needed to understand the duration of antibody production and how concentrations of antibodies change with time.

## Supplementary Information

Supplementary Information.

Supplementary Figure 1.

Supplementary Figure 2.
