# Supplementary material for: Detection of persistent SARS-CoV-2 IgG antibodies in oral mucosal fluid and upper respiratory tract specimens following COVID-19 mRNA vaccination
Source: Sci Rep. 2021 Dec 27;11:24448. doi: 10.1038/s41598-021-03931-3 (PMC8712521; doi:10.1038/s41598-021-03931-3)
Supplement: Supplementary file 1 — Supplementary Information. [file 41598_2021_3931_MOESM1_ESM.docx]

**Supplemental Materials**

*Methods for comparison study*

Each collection device was weighed prior to and following specimen collection to measure the volume of sample collected. The dilution factor of each specimen was calculated by dividing the volume of preservative fluid (800 µL) by the volume of the sample. The final concentration of SARS-CoV-2 IgG antibodies was determined by multiplying the specimen’s determined antibody concentration by the calculated dilution factor. Antibody concentration for other specimens was not adjusted for the dilution factor.

Between April 2020 and December 2020, we collected oral fluid specimens using the OSCD from participants who were previously infected with SARS-CoV-2 in a separate study cohort over time spanning a six-month period. Here, we determined titers of SARS-CoV-2 IgG antibodies in these previously collected oral fluid specimens.
