# Supplementary material for: Detection of persistent SARS-CoV-2 IgG antibodies in oral mucosal fluid and upper respiratory tract specimens following COVID-19 mRNA vaccination
Source: Sci Rep. 2021 Dec 27;11:24448. doi: 10.1038/s41598-021-03931-3 (PMC8712521; doi:10.1038/s41598-021-03931-3)
Supplement: Supplementary file 2 — Supplementary Figure 1. [file 41598_2021_3931_MOESM2_ESM.pdf]

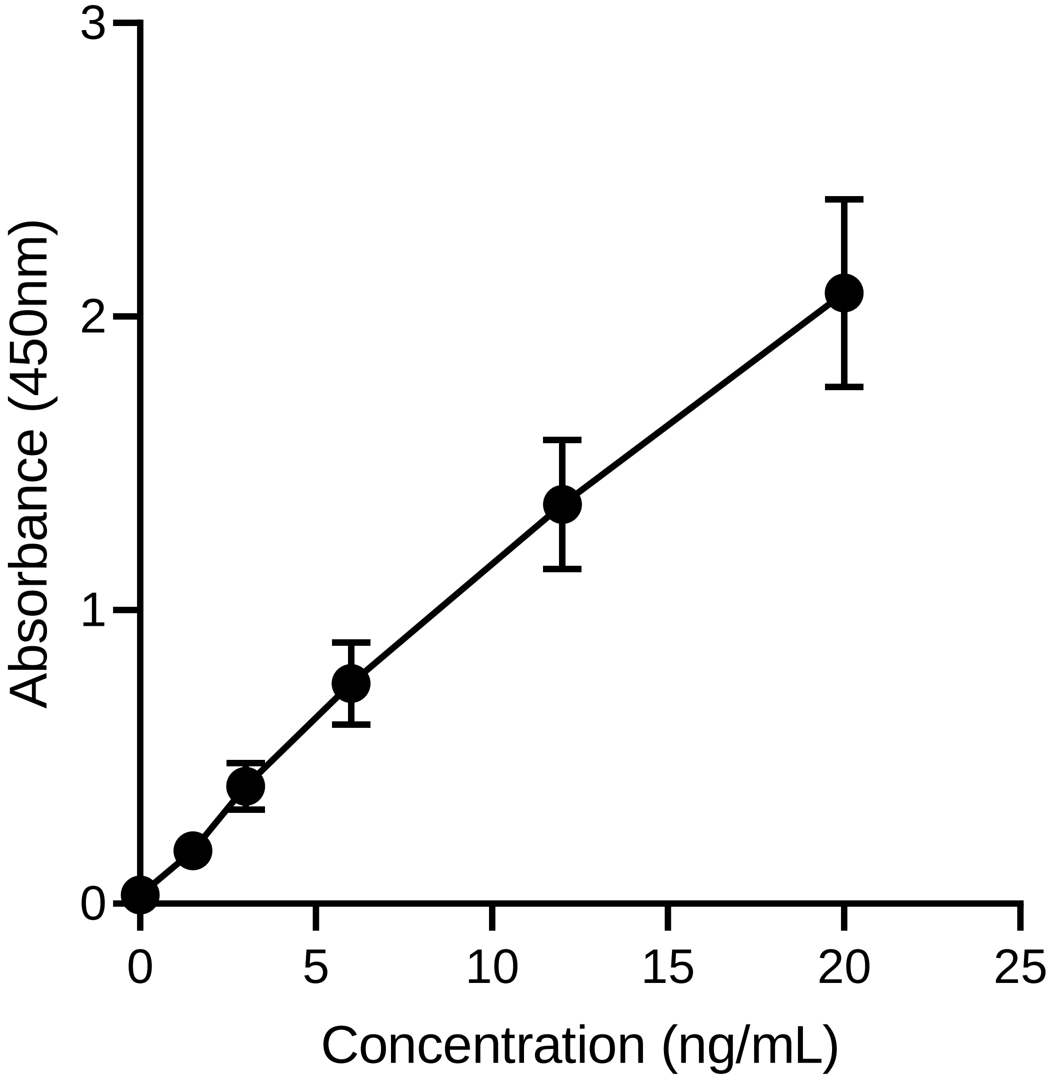

Supplementary Figure 1. Development of a standard curve for relative antibody quantification. Monoclonal IgG targeting the S1 antigen of SARS-CoV-2 was used to develop a quantitative assay for antibody quantification. The standard curve was developed based on monoclonal IgG antibody concentrations of 0ng/mL, 1.5ng/mL, 3ng/mL, 6ng/mL, 12ng/mL and 20ng/mL. The expression obtained from this standard curve, $y = 1.28x^2 + 6.89x + 0.137$, was used to determine sample concentrations from optical density (O.D.) measured at 450nm.
