# Supplementary material for: Detection of persistent SARS-CoV-2 IgG antibodies in oral mucosal fluid and upper respiratory tract specimens following COVID-19 mRNA vaccination
Source: Sci Rep. 2021 Dec 27;11:24448. doi: 10.1038/s41598-021-03931-3 (PMC8712521; doi:10.1038/s41598-021-03931-3)
Supplement: Supplementary file 3 — Supplementary Figure 2. [file 41598_2021_3931_MOESM3_ESM.pdf]

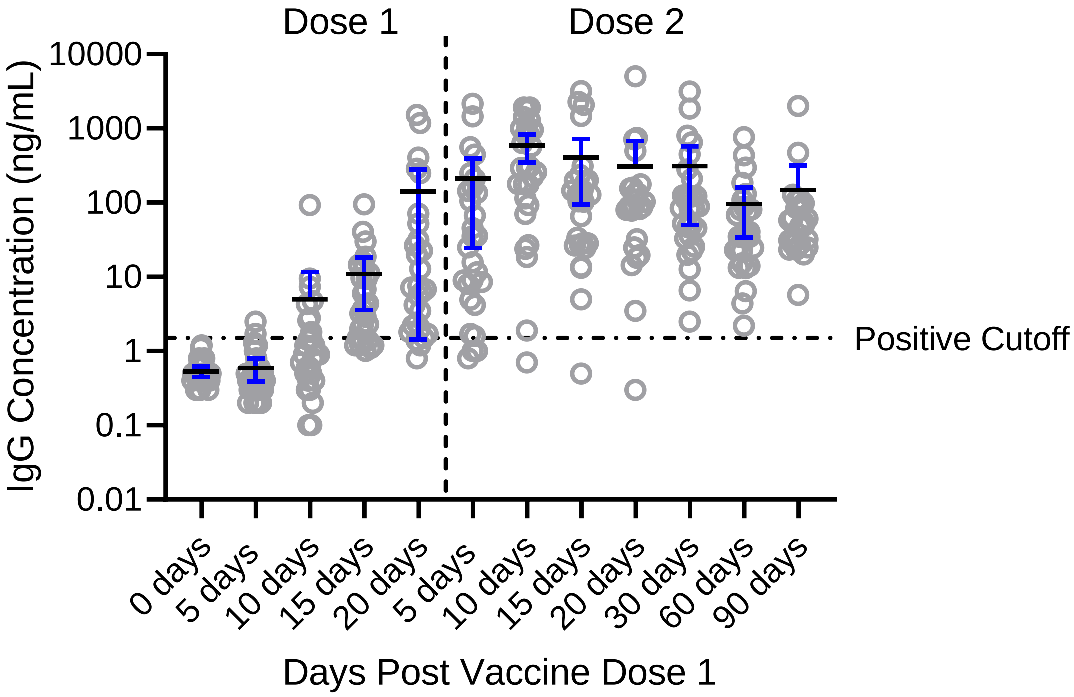

Supplementary Figure 2. SARS-CoV-2 IgG antibody concentration increase in nasal fluid post vaccination by the Pfizer vaccine. A subset of individuals in the Pfizer cohort (N=28) were also asked to contribute nasal swabs for relative antibody quantification. The time course trajectories of antibodies in nasal fluid were found to follow a similar trend to those in oral fluids.
